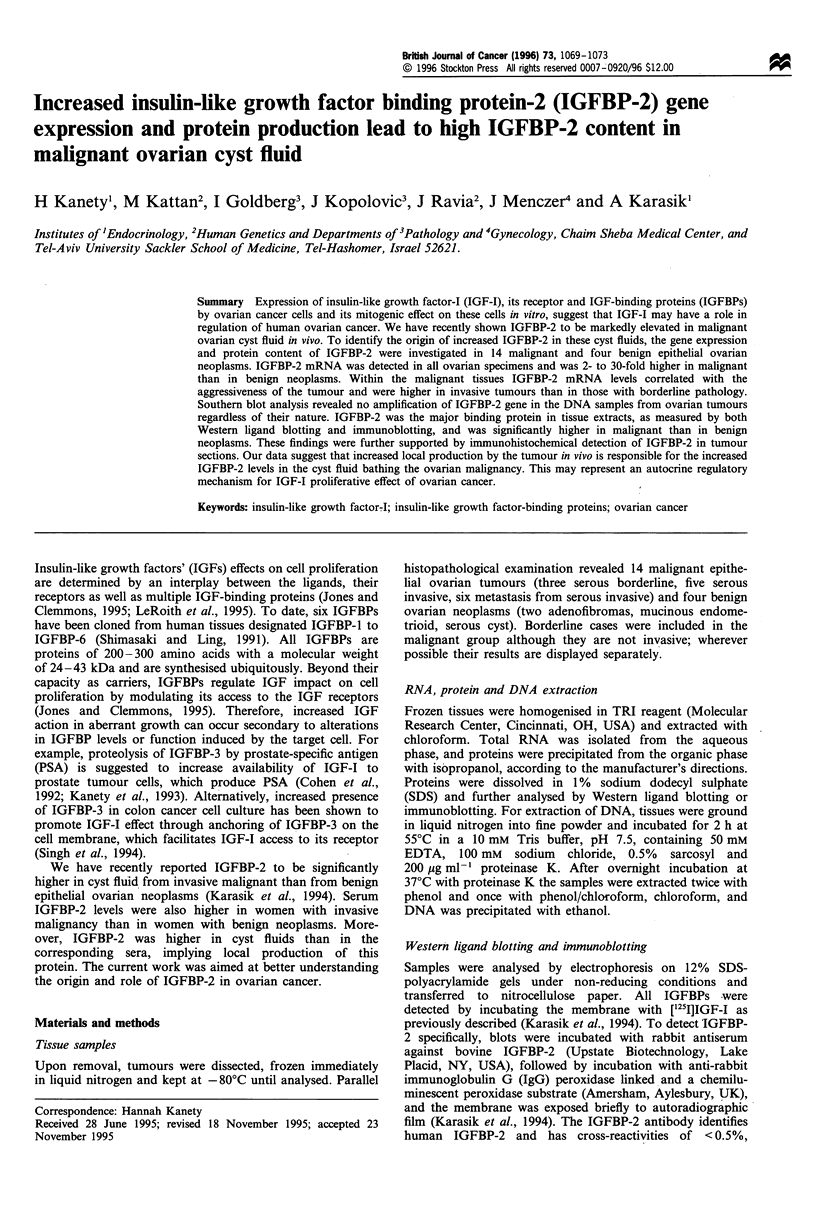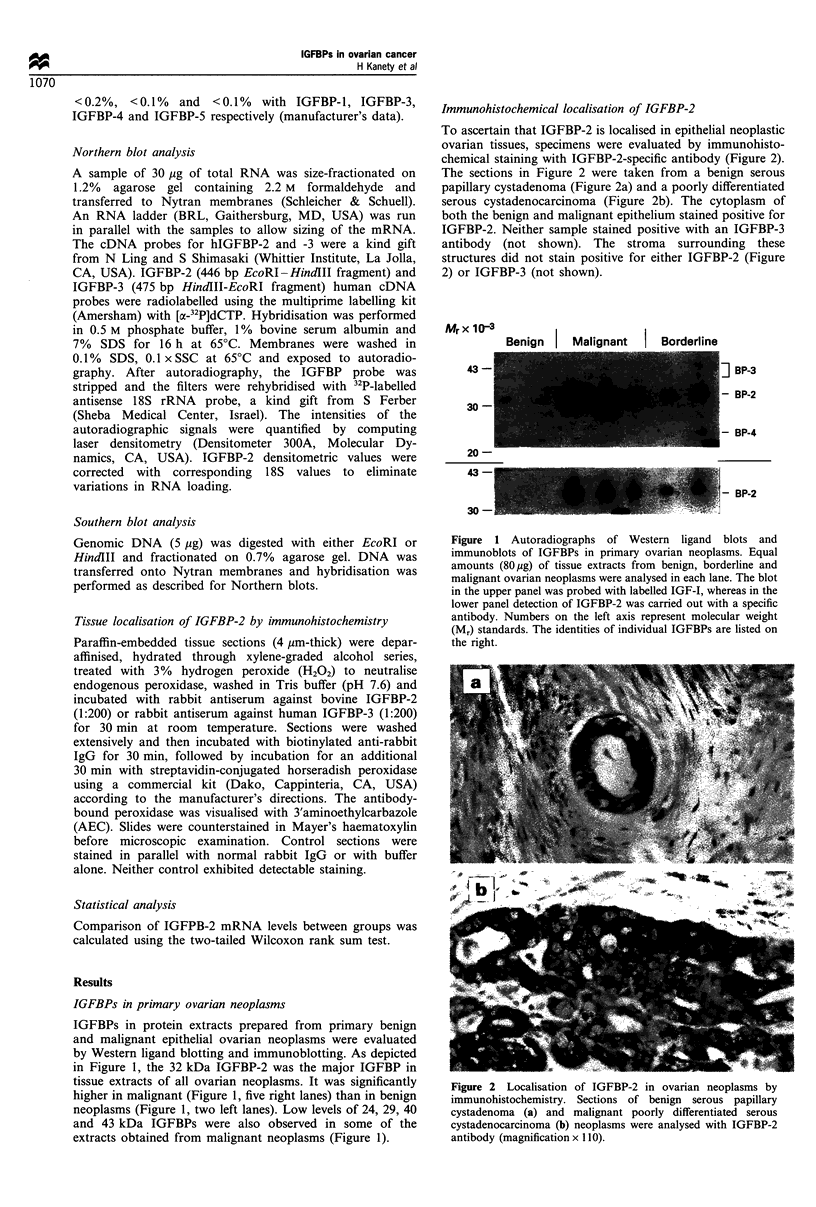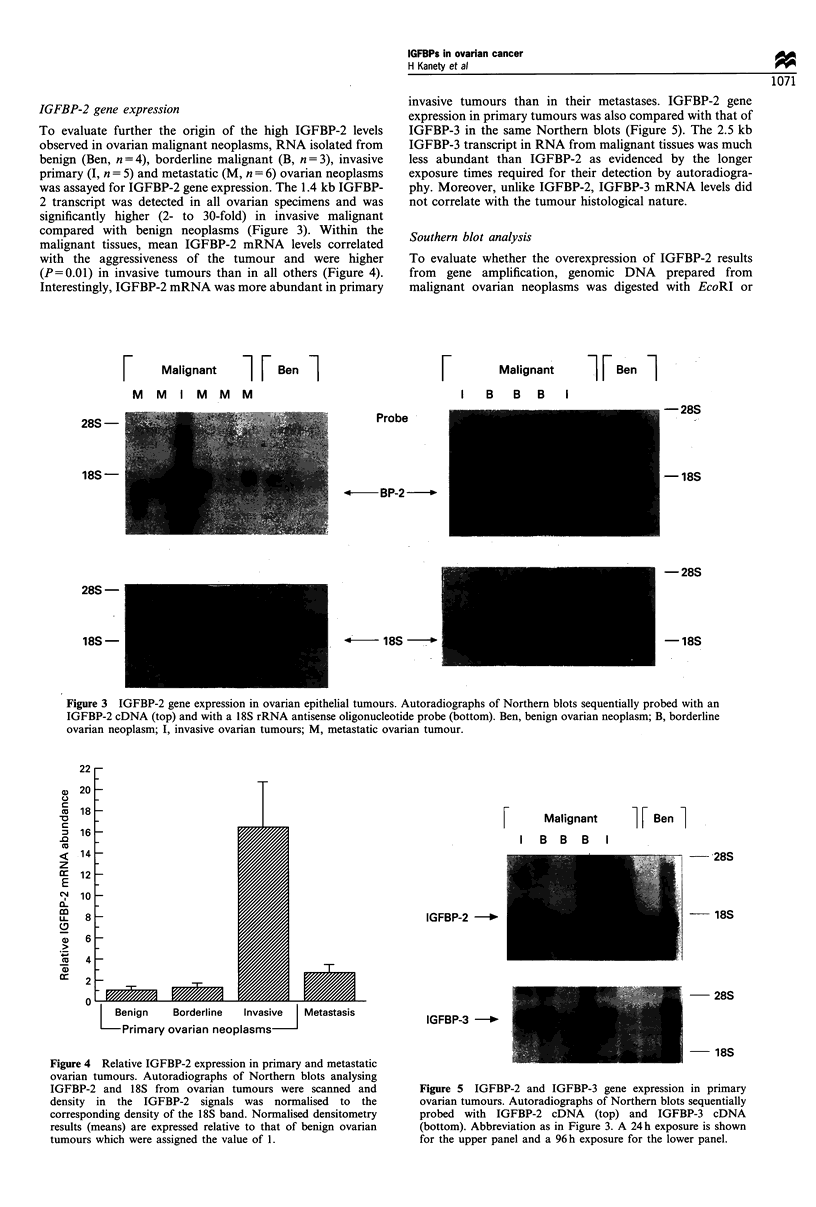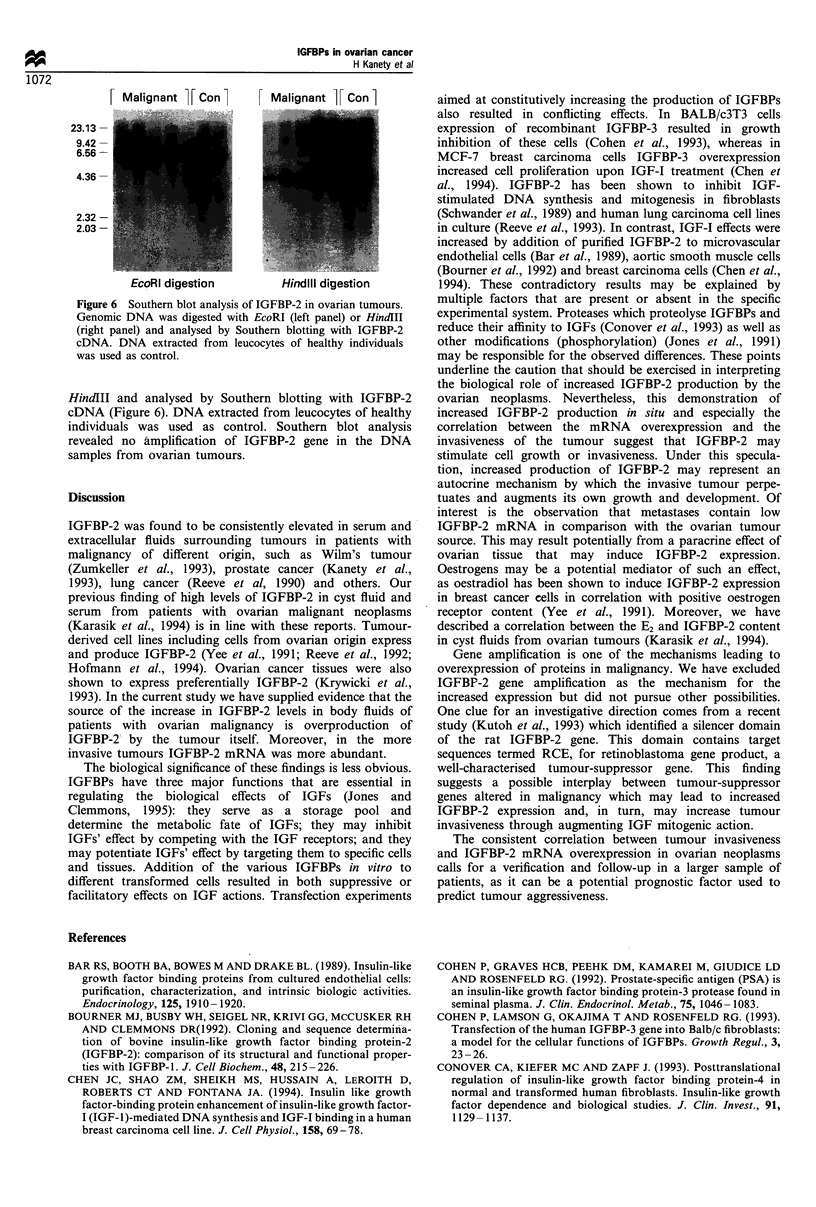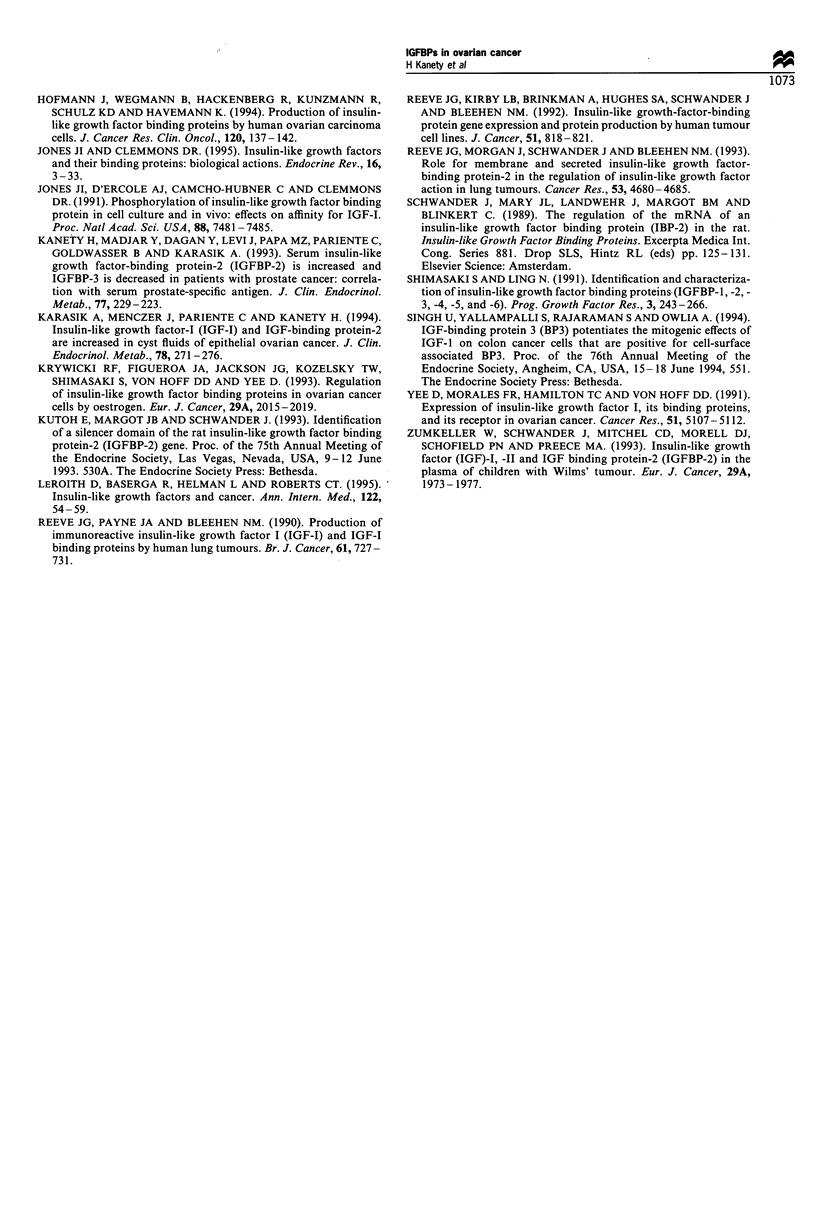# Increased insulin-like growth factor binding protein-2 (IGFBP-2) gene expression and protein production lead to high IGFBP-2 content in malignant ovarian cyst fluid.

**DOI:** 10.1038/bjc.1996.206

**Published:** 1996-05

**Authors:** H. Kanety, M. Kattan, I. Goldberg, J. Kopolovic, J. Ravia, J. Menczer, A. Karasik

**Affiliations:** Institute of Endocrinology, Chaim Sheba Medical Center, Tel-Hashomer, Israel.

## Abstract

**Images:**


British Journal of Cancer (1996) 73, 1069-1073
© 1996 Stockton Press All rights reserved 0007-0920/96 $12.00

### Increased insulin-like growth factor binding protein-2 (IGFBP-2) gene expression and protein production lead to high IGFBP-2 content in malignant ovarian cyst fluid

H Kanety[1], M Kattan[2], I Goldberg[3], J Kopolovic[3], J Ravia[2], J Menczer[4] and A Karasik[1]

*Institutes of [1]Endocrinology, [2]Human Genetics and Departments of [3]Pathology and [4]Gynecology, Chaim Sheba Medical Center, and Tel-Aviv University Sackler School of Medicine, Tel-Hashomer, Israel 52621.*

**Summary** Expression of insulin-like growth factor-I (IGF-I), its receptor and IGF-binding proteins (IGFBPs) by ovarian cancer cells and its mitogenic effect on these cells *in vitro*, suggest that IGF-I may have a role in regulation of human ovarian cancer. We have recently shown IGFBP-2 to be markedly elevated in malignant ovarian cyst fluid *in vivo*. To identify the origin of increased IGFBP-2 in these cyst fluids, the gene expression and protein content of IGFBP-2 were investigated in 14 malignant and four benign epithelial ovarian neoplasms. IGFBP-2 mRNA was detected in all ovarian specimens and was 2- to 30-fold higher in malignant than in benign neoplasms. Within the malignant tissues IGFBP-2 mRNA levels correlated with the aggressiveness of the tumour and were higher in invasive tumours than in those with borderline pathology. Southern blot analysis revealed no amplification of IGFBP-2 gene in the DNA samples from ovarian tumours regardless of their nature. IGFBP-2 was the major binding protein in tissue extracts, as measured by both Western ligand blotting and immunoblotting, and was significantly higher in malignant than in benign neoplasms. These findings were further supported by immunohistochemical detection of IGFBP-2 in tumour sections. Our data suggest that increased local production by the tumour *in vivo* is responsible for the increased IGFBP-2 levels in the cyst fluid bathing the ovarian malignancy. This may represent an autocrine regulatory mechanism for IGF-I proliferative effect of ovarian cancer.

**Keywords:** insulin-like growth factor-I; insulin-like growth factor-binding proteins; ovarian cancer

Insulin-like growth factors' (IGFs) effects on cell proliferation are determined by an interplay between the ligands, their receptors as well as multiple IGF-binding proteins (Jones and Clemmons, 1995; LeRoith *et al.*, 1995). To date, six IGFBPs have been cloned from human tissues designated IGFBP-1 to IGFBP-6 (Shimasaki and Ling, 1991). All IGFBPs are proteins of 200-300 amino acids with a molecular weight of 24-43 kDa and are synthesised ubiquitously. Beyond their capacity as carriers, IGFBPs regulate IGF impact on cell proliferation by modulating its access to the IGF receptors (Jones and Clemmons, 1995). Therefore, increased IGF action in aberrant growth can occur secondary to alterations in IGFBP levels or function induced by the target cell. For example, proteolysis of IGFBP-3 by prostate-specific antigen (PSA) is suggested to increase availability of IGF-I to prostate tumour cells, which produce PSA (Cohen *et al.*, 1992; Kanety *et al.*, 1993). Alternatively, increased presence of IGFBP-3 in colon cancer cell culture has been shown to promote IGF-I effect through anchoring of IGFBP-3 on the cell membrane, which facilitates IGF-I access to its receptor (Singh *et al.*, 1994).

We have recently reported IGFBP-2 to be significantly higher in cyst fluid from invasive malignant than from benign epithelial ovarian neoplasms (Karasik *et al.*, 1994). Serum IGFBP-2 levels were also higher in women with invasive malignancy than in women with benign neoplasms. Moreover, IGFBP-2 was higher in cyst fluids than in the corresponding sera, implying local production of this protein. The current work was aimed at better understanding the origin and role of IGFBP-2 in ovarian cancer.

### Materials and methods

### *Tissue samples*

Upon removal, tumours were dissected, frozen immediately in liquid nitrogen and kept at $-80°C$ until analysed. Parallel histopathological examination revealed 14 malignant epithelial ovarian tumours (three serous borderline, five serous invasive, six metastasis from serous invasive) and four benign ovarian neoplasms (two adenofibromas, mucinous endometrioid, serous cyst). Borderline cases were included in the malignant group although they are not invasive; wherever possible their results are displayed separately.

### *RNA, protein and DNA extraction*

Frozen tissues were homogenised in TRI reagent (Molecular Research Center, Cincinnati, OH, USA) and extracted with chloroform. Total RNA was isolated from the aqueous phase, and proteins were precipitated from the organic phase with isopropanol, according to the manufacturer's directions. Proteins were dissolved in 1% sodium dodecyl sulphate (SDS) and further analysed by Western ligand blotting or immunoblotting. For extraction of DNA, tissues were ground in liquid nitrogen into fine powder and incubated for 2 h at 55°C in a 10 mM Tris buffer, pH 7.5, containing 50 mM EDTA, 100 mM sodium chloride, 0.5% sarcosyl and 200 $\mu g\ ml^{-1}$ proteinase K. After overnight incubation at 37°C with proteinase K the samples were extracted twice with phenol and once with phenol/chloroform, chloroform, and DNA was precipitated with ethanol.

### *Western ligand blotting and immunoblotting*

Samples were analysed by electrophoresis on 12% SDS-polyacrylamide gels under non-reducing conditions and transferred to nitrocellulose paper. All IGFBPs were detected by incubating the membrane with [$^{125}$I]IGF-I as previously described (Karasik *et al.*, 1994). To detect IGFBP-2 specifically, blots were incubated with rabbit antiserum against bovine IGFBP-2 (Upstate Biotechnology, Lake Placid, NY, USA), followed by incubation with anti-rabbit immunoglobulin G (IgG) peroxidase linked and a chemiluminescent peroxidase substrate (Amersham, Aylesbury, UK), and the membrane was exposed briefly to autoradiographic film (Karasik *et al.*, 1994). The IGFBP-2 antibody identifies human IGFBP-2 and has cross-reactivities of <0.5%,

Correspondence: Hannah Kanety
Received 28 June 1995; revised 18 November 1995; accepted 23 November 1995

$<0.2\%$, $<0.1\%$ and $<0.1\%$ with IGFBP-1, IGFBP-3, IGFBP-4 and IGFBP-5 respectively (manufacturer's data).

*Northern blot analysis*

A sample of 30 $\mu g$ of total RNA was size-fractionated on 1.2% agarose gel containing 2.2 M formaldehyde and transferred to Nytran membranes (Schleicher & Schuell). An RNA ladder (BRL, Gaithersburg, MD, USA) was run in parallel with the samples to allow sizing of the mRNA. The cDNA probes for hIGFBP-2 and -3 were a kind gift from N Ling and S Shimasaki (Whittier Institute, La Jolla, CA, USA). IGFBP-2 (446 bp *Eco*RI–*Hind*III fragment) and IGFBP-3 (475 bp *Hind*III-*Eco*RI fragment) human cDNA probes were radiolabelled using the multiprime labelling kit (Amersham) with [$\alpha$-$^{32}$P]dCTP. Hybridisation was performed in 0.5 M phosphate buffer, 1% bovine serum albumin and 7% SDS for 16 h at 65°C. Membranes were washed in 0.1% SDS, 0.1 × SSC at 65°C and exposed to autoradiography. After autoradiography, the IGFBP probe was stripped and the filters were rehybridised with $^{32}$P-labelled antisense 18S rRNA probe, a kind gift from S Ferber (Sheba Medical Center, Israel). The intensities of the autoradiographic signals were quantified by computing laser densitometry (Densitometer 300A, Molecular Dynamics, CA, USA). IGFBP-2 densitometric values were corrected with corresponding 18S values to eliminate variations in RNA loading.

*Southern blot analysis*

Genomic DNA (5 $\mu g$) was digested with either *Eco*RI or *Hind*III and fractionated on 0.7% agarose gel. DNA was transferred onto Nytran membranes and hybridisation was performed as described for Northern blots.

*Tissue localisation of IGFBP-2 by immunohistochemistry*

Paraffin-embedded tissue sections (4 $\mu$m-thick) were deparaffinised, hydrated through xylene-graded alcohol series, treated with 3% hydrogen peroxide ($H_2O_2$) to neutralise endogenous peroxidase, washed in Tris buffer (pH 7.6) and incubated with rabbit antiserum against bovine IGFBP-2 (1:200) or rabbit antiserum against human IGFBP-3 (1:200) for 30 min at room temperature. Sections were washed extensively and then incubated with biotinylated anti-rabbit IgG for 30 min, followed by incubation for an additional 30 min with streptavidin-conjugated horseradish peroxidase using a commercial kit (Dako, Cappinteria, CA, USA) according to the manufacturer's directions. The antibody-bound peroxidase was visualised with 3′aminoethylcarbazole (AEC). Slides were counterstained in Mayer's haematoxylin before microscopic examination. Control sections were stained in parallel with normal rabbit IgG or with buffer alone. Neither control exhibited detectable staining.

*Statistical analysis*

Comparison of IGFPB-2 mRNA levels between groups was calculated using the two-tailed Wilcoxon rank sum test.

### Results

*IGFBPs in primary ovarian neoplasms*

IGFBPs in protein extracts prepared from primary benign and malignant epithelial ovarian neoplasms were evaluated by Western ligand blotting and immunoblotting. As depicted in Figure 1, the 32 kDa IGFBP-2 was the major IGFBP in tissue extracts of all ovarian neoplasms. It was significantly higher in malignant (Figure 1, five right lanes) than in benign neoplasms (Figure 1, two left lanes). Low levels of 24, 29, 40 and 43 kDa IGFBPs were also observed in some of the extracts obtained from malignant neoplasms (Figure 1).

*Immunohistochemical localisation of IGFBP-2*

To ascertain that IGFBP-2 is localised in epithelial neoplastic ovarian tissues, specimens were evaluated by immunohistochemical staining with IGFBP-2-specific antibody (Figure 2). The sections in Figure 2 were taken from a benign serous papillary cystadenoma (Figure 2a) and a poorly differentiated serous cystadenocarcinoma (Figure 2b). The cytoplasm of both the benign and malignant epithelium stained positive for IGFBP-2. Neither sample stained positive with an IGFBP-3 antibody (not shown). The stroma surrounding these structures did not stain positive for either IGFBP-2 (Figure 2) or IGFBP-3 (not shown).

**Figure 1** Autoradiographs of Western ligand blots and immunoblots of IGFBPs in primary ovarian neoplasms. Equal amounts (80 $\mu g$) of tissue extracts from benign, borderline and malignant ovarian neoplasms were analysed in each lane. The blot in the upper panel was probed with labelled IGF-I, whereas in the lower panel detection of IGFBP-2 was carried out with a specific antibody. Numbers on the left axis represent molecular weight ($M_r$) standards. The identities of individual IGFBPs are listed on the right.

**Figure 2** Localisation of IGFBP-2 in ovarian neoplasms by immunohistochemistry. Sections of benign serous papillary cystadenoma (**a**) and malignant poorly differentiated serous cystadenocarcinoma (**b**) neoplasms were analysed with IGFBP-2 antibody (magnification × 110).

*IGFBP-2 gene expression*

To evaluate further the origin of the high IGFBP-2 levels observed in ovarian malignant neoplasms, RNA isolated from benign (Ben, $n=4$), borderline malignant (B, $n=3$), invasive primary (I, $n=5$) and metastatic (M, $n=6$) ovarian neoplasms was assayed for IGFBP-2 gene expression. The 1.4 kb IGFBP-2 transcript was detected in all ovarian specimens and was significantly higher (2- to 30-fold) in invasive malignant compared with benign neoplasms (Figure 3). Within the malignant tissues, mean IGFBP-2 mRNA levels correlated with the aggressiveness of the tumour and were higher ($P=0.01$) in invasive tumours than in all others (Figure 4). Interestingly, IGFBP-2 mRNA was more abundant in primary invasive tumours than in their metastases. IGFBP-2 gene expression in primary tumours was also compared with that of IGFBP-3 in the same Northern blots (Figure 5). The 2.5 kb IGFBP-3 transcript in RNA from malignant tissues was much less abundant than IGFBP-2 as evidenced by the longer exposure times required for their detection by autoradiography. Moreover, unlike IGFBP-2, IGFBP-3 mRNA levels did not correlate with the tumour histological nature.

*Southern blot analysis*

To evaluate whether the overexpression of IGFBP-2 results from gene amplification, genomic DNA prepared from malignant ovarian neoplasms was digested with *Eco*RI or

**Figure 3** IGFBP-2 gene expression in ovarian epithelial tumours. Autoradiographs of Northern blots sequentially probed with an IGFBP-2 cDNA (top) and with a 18S rRNA antisense oligonucleotide probe (bottom). Ben, benign ovarian neoplasm; B, borderline ovarian neoplasm; I, invasive ovarian tumours; M, metastatic ovarian tumour.

**Figure 4** Relative IGFBP-2 expression in primary and metastatic ovarian tumours. Autoradiographs of Northern blots analysing IGFBP-2 and 18S from ovarian tumours were scanned and density in the IGFBP-2 signals was normalised to the corresponding density of the 18S band. Normalised densitometry results (means) are expressed relative to that of benign ovarian tumours which were assigned the value of 1.

**Figure 5** IGFBP-2 and IGFBP-3 gene expression in primary ovarian tumours. Autoradiographs of Northern blots sequentially probed with IGFBP-2 cDNA (top) and IGFBP-3 cDNA (bottom). Abbreviation as in Figure 3. A 24 h exposure is shown for the upper panel and a 96 h exposure for the lower panel.

**Figure 6** Southern blot analysis of IGFBP-2 in ovarian tumours. Genomic DNA was digested with *Eco*RI (left panel) or *Hind*III (right panel) and analysed by Southern blotting with IGFBP-2 cDNA. DNA extracted from leucocytes of healthy individuals was used as control.

*Hind*III and analysed by Southern blotting with IGFBP-2 cDNA (Figure 6). DNA extracted from leucocytes of healthy individuals was used as control. Southern blot analysis revealed no amplification of IGFBP-2 gene in the DNA samples from ovarian tumours.

### Discussion

IGFBP-2 was found to be consistently elevated in serum and extracellular fluids surrounding tumours in patients with malignancy of different origin, such as Wilm's tumour (Zumkeller *et al.*, 1993), prostate cancer (Kanety *et al.*, 1993), lung cancer (Reeve *et al*, 1990) and others. Our previous finding of high levels of IGFBP-2 in cyst fluid and serum from patients with ovarian malignant neoplasms (Karasik *et al.*, 1994) is in line with these reports. Tumour-derived cell lines including cells from ovarian origin express and produce IGFBP-2 (Yee *et al.*, 1991; Reeve *et al.*, 1992; Hofmann *et al.*, 1994). Ovarian cancer tissues were also shown to express preferentially IGFBP-2 (Krywicki *et al.*, 1993). In the current study we have supplied evidence that the source of the increase in IGFBP-2 levels in body fluids of patients with ovarian malignancy is overproduction of IGFBP-2 by the tumour itself. Moreover, in the more invasive tumours IGFBP-2 mRNA was more abundant.

The biological significance of these findings is less obvious. IGFBPs have three major functions that are essential in regulating the biological effects of IGFs (Jones and Clemmons, 1995): they serve as a storage pool and determine the metabolic fate of IGFs; they may inhibit IGFs' effect by competing with the IGF receptors; and they may potentiate IGFs' effect by targeting them to specific cells and tissues. Addition of the various IGFBPs *in vitro* to different transformed cells resulted in both suppressive or facilitatory effects on IGF actions. Transfection experiments aimed at constitutively increasing the production of IGFBPs also resulted in conflicting effects. In BALB/c3T3 cells expression of recombinant IGFBP-3 resulted in growth inhibition of these cells (Cohen *et al.*, 1993), whereas in MCF-7 breast carcinoma cells IGFBP-3 overexpression increased cell proliferation upon IGF-I treatment (Chen *et al.*, 1994). IGFBP-2 has been shown to inhibit IGF-stimulated DNA synthesis and mitogenesis in fibroblasts (Schwander *et al.*, 1989) and human lung carcinoma cell lines in culture (Reeve *et al.*, 1993). In contrast, IGF-I effects were increased by addition of purified IGFBP-2 to microvascular endothelial cells (Bar *et al.*, 1989), aortic smooth muscle cells (Bourner *et al.*, 1992) and breast carcinoma cells (Chen *et al.*, 1994). These contradictory results may be explained by multiple factors that are present or absent in the specific experimental system. Proteases which proteolyse IGFBPs and reduce their affinity to IGFs (Conover *et al.*, 1993) as well as other modifications (phosphorylation) (Jones *et al.*, 1991) may be responsible for the observed differences. These points underline the caution that should be exercised in interpreting the biological role of increased IGFBP-2 production by the ovarian neoplasms. Nevertheless, this demonstration of increased IGFBP-2 production *in situ* and especially the correlation between the mRNA overexpression and the invasiveness of the tumour suggest that IGFBP-2 may stimulate cell growth or invasiveness. Under this speculation, increased production of IGFBP-2 may represent an autocrine mechanism by which the invasive tumour perpetuates and augments its own growth and development. Of interest is the observation that metastases contain low IGFBP-2 mRNA in comparison with the ovarian tumour source. This may result potentially from a paracrine effect of ovarian tissue that may induce IGFBP-2 expression. Oestrogens may be a potential mediator of such an effect, as oestradiol has been shown to induce IGFBP-2 expression in breast cancer cells in correlation with positive oestrogen receptor content (Yee *et al.*, 1991). Moreover, we have described a correlation between the $E_2$ and IGFBP-2 content in cyst fluids from ovarian tumours (Karasik *et al.*, 1994).

Gene amplification is one of the mechanisms leading to overexpression of proteins in malignancy. We have excluded IGFBP-2 gene amplification as the mechanism for the increased expression but did not pursue other possibilities. One clue for an investigative direction comes from a recent study (Kutoh *et al.*, 1993) which identified a silencer domain of the rat IGFBP-2 gene. This domain contains target sequences termed RCE, for retinoblastoma gene product, a well-characterised tumour-suppressor gene. This finding suggests a possible interplay between tumour-suppressor genes altered in malignancy which may lead to increased IGFBP-2 expression and, in turn, may increase tumour invasiveness through augmenting IGF mitogenic action.

The consistent correlation between tumour invasiveness and IGFBP-2 mRNA overexpression in ovarian neoplasms calls for a verification and follow-up in a larger sample of patients, as it can be a potential prognostic factor used to predict tumour aggressiveness.

### References

BAR RS, BOOTH BA, BOWES M AND DRAKE BL. (1989). Insulin-like growth factor binding proteins from cultured endothelial cells: purification, characterization, and intrinsic biologic activities. *Endocrinology*, **125,** 1910–1920.

BOURNER MJ, BUSBY WH, SEIGEL NR, KRIVI GG, MCCUSKER RH AND CLEMMONS DR(1992). Cloning and sequence determination of bovine insulin-like growth factor binding protein-2 (IGFBP-2): comparison of its structural and functional properties with IGFBP-1. *J. Cell Biochem.*, **48,** 215–226.

CHEN JC, SHAO ZM, SHEIKH MS, HUSSAIN A, LEROITH D, ROBERTS CT AND FONTANA JA. (1994). Insulin like growth factor-binding protein enhancement of insulin-like growth factor-I (IGF-1)-mediated DNA synthesis and IGF-I binding in a human breast carcinoma cell line. *J. Cell Physiol.*, **158,** 69–78.

COHEN P, GRAVES HCB, PEEHK DM, KAMAREI M, GIUDICE LD AND ROSENFELD RG. (1992). Prostate-specific antigen (PSA) is an insulin-like growth factor binding protein-3 protease found in seminal plasma. *J. Clin. Endocrinol. Metab.*, **75,** 1046–1083.

COHEN P, LAMSON G, OKAJIMA T AND ROSENFELD RG. (1993). Transfection of the human IGFBP-3 gene into Balb/c fibroblasts: a model for the cellular functions of IGFBPs. *Growth Regul.*, **3,** 23–26.

CONOVER CA, KIEFER MC AND ZAPF J. (1993). Posttranslational regulation of insulin-like growth factor binding protein-4 in normal and transformed human fibroblasts. Insulin-like growth factor dependence and biological studies. *J. Clin. Invest.*, **91,** 1129–1137.

HOFMANN J, WEGMANN B, HACKENBERG R, KUNZMANN R, SCHULZ KD AND HAVEMANN K. (1994). Production of insulin-like growth factor binding proteins by human ovarian carcinoma cells. *J. Cancer Res. Clin. Oncol.*, **120,** 137–142.

JONES JI AND CLEMMONS DR. (1995). Insulin-like growth factors and their binding proteins: biological actions. *Endocrine Rev.*, **16,** 3–33.

JONES JI, D'ERCOLE AJ, CAMCHO-HUBNER C AND CLEMMONS DR. (1991). Phosphorylation of insulin-like growth factor binding protein in cell culture and in vivo: effects on affinity for IGF-I. *Proc. Natl Acad. Sci. USA*, **88,** 7481–7485.

KANETY H, MADJAR Y, DAGAN Y, LEVI J, PAPA MZ, PARIENTE C, GOLDWASSER B AND KARASIK A. (1993). Serum insulin-like growth factor-binding protein-2 (IGFBP-2) is increased and IGFBP-3 is decreased in patients with prostate cancer: correlation with serum prostate-specific antigen. *J. Clin. Endocrinol. Metab.*, **77,** 229–223.

KARASIK A, MENCZER J, PARIENTE C AND KANETY H. (1994). Insulin-like growth factor-I (IGF-I) and IGF-binding protein-2 are increased in cyst fluids of epithelial ovarian cancer. *J. Clin. Endocrinol. Metab.*, **78,** 271–276.

KRYWICKI RF, FIGUEROA JA, JACKSON JG, KOZELSKY TW, SHIMASAKI S, VON HOFF DD AND YEE D. (1993). Regulation of insulin-like growth factor binding proteins in ovarian cancer cells by oestrogen. *Eur. J. Cancer*, **29A,** 2015–2019.

KUTOH E, MARGOT JB AND SCHWANDER J. (1993). Identification of a silencer domain of the rat insulin-like growth factor binding protein-2 (IGFBP-2) gene. Proc. of the 75th Annual Meeting of the Endocrine Society, Las Vegas, Nevada, USA, 9–12 June 1993. 530A. The Endocrine Society Press: Bethesda.

LEROITH D, BASERGA R, HELMAN L AND ROBERTS CT. (1995). Insulin-like growth factors and cancer. *Ann. Intern. Med.*, **122,** 54–59.

REEVE JG, PAYNE JA AND BLEEHEN NM. (1990). Production of immunoreactive insulin-like growth factor I (IGF-I) and IGF-I binding proteins by human lung tumours. *Br. J. Cancer*, **61,** 727–731.

REEVE JG, KIRBY LB, BRINKMAN A, HUGHES SA, SCHWANDER J AND BLEEHEN NM. (1992). Insulin-like growth-factor-binding protein gene expression and protein production by human tumour cell lines. *J. Cancer*, **51,** 818–821.

REEVE JG, MORGAN J, SCHWANDER J AND BLEEHEN NM. (1993). Role for membrane and secreted insulin-like growth factor-binding protein-2 in the regulation of insulin-like growth factor action in lung tumours. *Cancer Res.*, **53,** 4680–4685.

SCHWANDER J, MARY JL, LANDWEHR J, MARGOT BM AND BLINKERT C. (1989). The regulation of the mRNA of an insulin-like growth factor binding protein (IBP-2) in the rat. *Insulin-like Growth Factor Binding Proteins.* Excerpta Medica Int. Cong. Series 881. Drop SLS, Hintz RL (eds) pp. 125–131. Elsevier Science: Amsterdam.

SHIMASAKI S AND LING N. (1991). Identification and characterization of insulin-like growth factor binding proteins (IGFBP-1, -2, -3, -4, -5, and -6). *Prog. Growth Factor Res.*, **3,** 243–266.

SINGH U, YALLAMPALLI S, RAJARAMAN S AND OWLIA A. (1994). IGF-binding protein 3 (BP3) potentiates the mitogenic effects of IGF-1 on colon cancer cells that are positive for cell-surface associated BP3. Proc. of the 76th Annual Meeting of the Endocrine Society, Angheim, CA, USA, 15–18 June 1994, 551. The Endocrine Society Press: Bethesda.

YEE D, MORALES FR, HAMILTON TC AND VON HOFF DD. (1991). Expression of insulin-like growth factor I, its binding proteins, and its receptor in ovarian cancer. *Cancer Res.*, **51,** 5107–5112.

ZUMKELLER W, SCHWANDER J, MITCHEL CD, MORELL DJ, SCHOFIELD PN AND PREECE MA. (1993). Insulin-like growth factor (IGF)-I, -II and IGF binding protein-2 (IGFBP-2) in the plasma of children with Wilms' tumour. *Eur. J. Cancer*, **29A,** 1973–1977.